# Physical exercise and liver “fitness”: Role of mitochondrial function and epigenetics-related mechanisms in non-alcoholic fatty liver disease

**DOI:** 10.1016/j.molmet.2019.11.015

**Published:** 2019-11-29

**Authors:** Jelena Stevanović, Jorge Beleza, Pedro Coxito, António Ascensão, José Magalhães

**Affiliations:** 1 Laboratory of Metabolism and Exercise (LaMetEx), Research Centre in Physical Activity, Health and Leisure (CIAFEL), Faculty of Sport, University of Porto, Porto, Portugal; 2 Department of Cell Biology, Physiology & Immunology, Faculty of Biology, University of Barcelona, Barcelona, Spain

**Keywords:** NAFLD, Physical exercise, Mitochondrial dysfunction, Epigenetics

## Abstract

**Background:**

Modern lifestyles, especially high-caloric intake and physical inactivity, contribute to the increased prevalence of non-alcoholic fatty liver disease (NAFLD), which becomes a significant health problem worldwide. Lifestyle changes, however, affect not only parental generation, but also their offspring, reinforcing the need for efficient preventive approaches to deal with this disease. This transgenerational influence of phenotypes dependent on parents (particularly maternal) behaviours may open additional research avenues. Despite persistent attempts to design an effective pharmacological therapy against NAFLD, physical activity, as a non-pharmacological approach, emerges as an exciting strategy.

**Scope of Review:**

Here we briefly review the effect of physical exercise on liver mitochondria adaptations in NAFLD, highlighting the importance of mitochondrial metabolism and transgenerational and epigenetic mechanisms in liver diseases.

**Major Conclusions:**

A deeper look into cellular mechanisms sheds a light on possible effects of physical activity in the prevention and treatment of NAFLD through modulation of function and structure of particular organelles, namely mitochondria. Additionally, despite of increasing evidence regarding the contribution of epigenetic mechanisms in the pathogenesis of different diseases, the role of microRNAs, DNA methylation, and histone modification in NAFLD pathogenesis still needs to be elucidated.

## Highlights

• Modern lifestyle, including physical inactivity, contribute to NAFLD development.
• Maternal lifestyle through epigenetic mechanisms may influence offspring health.
• Exercise is a potential protective non-pharmacological strategy against NAFLD.
• Exercise can modulate liver mitochondrial structure and function.
• Exercise-related epigenetic mechanisms may explain exercise role in NAFLD treatment.

## Abbreviations

Drp1: dynamin-related protein 1
ER: estrogen receptor
ERE: estrogen response elements
ET: endurance training
ETC: electron transport chain
FFA: free fatty acid
Fgf21: fibroblast growth factor-21
GCKR: glucokinase regulatory protein
Fis1: human fission factor-1
GDM: gestational diabetes mellitus
HFD: high-fat diet
IMM: inner mitochondrial membranes
IR: insulin resistance
LYPLAL1: lysophospholipase-like-1
MBOAT7: membrane bound O-acyltransferase domain-containing-7
MFF: mitochondria fission factor
Mfn1: mitofusin1
Mfn2: mitofusin2
MiD49: mitochondrial dynamics protein of 49 kDa
MiD51: mitochondrial dynamics protein of 51 kDa
miRNA: microRNA
mPTP: mitochondrial permeability transition pores
NADH: dihydro-nicotinamide adenine dinucleotide
NAFLD: non-alcoholic fatty liver disease
NASH: non-alcoholic steatohepatitis
OMM: outer mitochondrial membranes
OPA1: optic atrophy 1
OXPHOS: oxidative phosphorylation
PGC1α: peroxisome proliferators-activated receptor-gamma coactivator 1-alpha
PNPLA3: patatin-like phospholipase domain-containing 3
PPAR: peroxisome proliferators-activated receptor
ROS: reactive oxygen species
T2DM: type 2 diabetes mellitus
TFAM: mitochondrial transcription factor A
TG: triglycerides
TM6SF2: transmembrane 6 superfamily member-2
UCP2: uncoupling protein-2
VLDL: very low-density lipoproteins
VPA: voluntary physical activity

## 1 Introduction

Sedentary lifestyle together with overconsumption of high-caloric diet has been identified as a major cause of metabolic diseases, including the one defined as non-alcoholic fatty liver disease (NAFLD). Usually described as the hepatic manifestation of metabolic syndrome, NAFLD includes a spectrum of conditions, from hepatic steatosis to non-alcoholic steatohepatitis (NASH) and cirrhosis, characterized by lipid accumulation in liver cells [[1], [2], [3]]. NAFLD is associated with common metabolic disorders, such as, obesity [4], increased cardiovascular risk [5,6], chronic kidney disease [7], insulin resistance (IR) and type 2 diabetes (T2DM) [8], but it has also been associated with obstructive sleep apnea, osteoporosis, psoriasis, periodontitis, hypothyroidism, male sexual dysfunction, polycystic ovarian syndrome, colonorectal cancer, and urolithiasis, proposing NAFLD as a metabolic disease with extra-hepatic manifestations and multi-organ involvement [9,10].

The global prevalence of NAFLD has increased in the last two decades and it is estimated to be about 25% with variations in prevalence in different regions of the world [11,12]. A meta-analytic study of NAFLD global epidemiology showed that the lowest prevalence of NAFLD is observed in Africa, and the highest in Middle East and South America. Moreover, this same study supports the association of metabolic comorbidities of NAFLD and obesity, T2DM, and metabolic syndrome in the same areas of the globe [11]. The NAFLD prevalence also increases with age, and it is more common in men than in women, in which the prevalence is greatly increased after menopause [[13], [14], [15]]. This gender-specific difference in NAFLD prevalence relies on the possible protective effect of sex hormones, especially estrogen, against the risk of developing NAFLD in women [15].

In this context, it is also important to note that lifestyle may affect not only the parental generation, but also the offspring resistance to develop chronic diseases later in life. Factors to which a mother is exposed during the pregnancy period, such as diet, stress, tobacco and alcohol consumption, or even gestational diabetes mellitus (GDM), may affect fetal development through epigenetic mechanisms that alter fetal phenotype [16,17]. Pregnancy is a period during which a woman's body undergoes multiple metabolic and physiological adaptations necessary to support the demands of pregnancy, as well as to provide physiological environment for fetus development. Changes in maternal lifestyle during pregnancy, especially changes in nutrition and physical activity, may affect those adaptations, and consequently, fetus growth and development [18,19]. Recent data from animal models show that maternal nutrition during pregnancy and lactation may affect the metabolic phenotype of the offspring, making them more prone to alterations in liver lipid metabolism and hepatic steatosis [[20], [21], [22], [23]].

Therefore, to understand the potential clinical and economic burden of NAFLD in the future, recent studies have shed light on possible therapeutic approaches to address this growing challenge. Still, to design the most effective therapy, it is necessary to recognize the underlying mechanisms in the development and progression of NAFLD. As an active metabolic tissue, the liver has important bioenergetics and xenobiotic detoxification roles, and the disruption of its function may lead to different pathological states [24]. Furthermore, given the pivotal role of mitochondrial network in metabolism and cellular energy production, regulation of ion homeostasis and redox signaling as well as in cellular remodeling and adaptation, dysfunction of liver mitochondria seems to have an important contribution to the development of liver diseases. In fact, liver mitochondrial degeneration has been associated with the hepatocyte death and inflammation, contributing to the degenerative processes. Moreover, mitochondria are recognized as important sources and targets of reactive oxygen species (ROS) associated with liver diseases [1]. They have a central role in cellular lipid metabolism and oxidative stress [25], with mitochondrial network dysfunction being proposed as one of the first events that occur during the development of NAFLD. The inability of mitochondria to coordinate subcellular metabolic processes in the liver results in the impaired regulation of hepatic lipid metabolism and consequent lipid accumulation in hepatocytes [[25], [26], [27]]. Recent data further suggest the important role of endoplasmic reticulum, an important organelle for calcium (Ca^2+^) homeostasis and lipid synthesis, in the pathogenesis of NAFLD. The disruption of endoplasmic reticulum homeostasis activates different signalling pathways related to NAFLD progression, including those involved in inflammation and apoptosis [[28], [29], [30]]. Furthermore, not only that the disturbance of mitochondria and endoplasmic reticulum homeostasis leads to signaling and metabolic changes, but also causes the loss of physical or functional interactions between these two extremely relevant organelles related to cellular metabolism. These tight contact points between mitochondria and endoplasmic reticulum, known as mitochondria-associated endoplasmic reticulum membranes (MAMs), are vital in the regulation of lipid transport, Ca^2+^ homeostasis, inflammatory signaling and cellular survival [31,32] and thus, the disruption of MAMs integrity is proposed to be involved in hepatic metabolic diseases, including NAFLD [33].

Despite the increased knowledge of underlying cellular mechanisms related to NAFLD, the design of a standard effective therapeutic approach for such complex and multifactorial disease seems to be out of reach so far. Pharmacological interventions and bariatric surgery are proposed in the treatment of NAFLD [34,35]; however, lifestyle modifications, particularly physical exercise, have been suggested as a first-line approach. Being considered one of the most relevant environmental risk factors in NAFLD development, it is no wonder that sedentary behavior is an important target in the NAFLD treatment [1,36]. Physical inactivity has been positively associated with the increased prevalence of a very significant number of chronic diseases and premature mortality. According to the World Health Organization (WHO), it is the 4th leading global risk for mortality, followed by overweight and obesity [37]. Around 30% of people worldwide are physically inactive, with women being slightly more inactive than men [38]. The latest Eurobarometer by the European Commission shows that only 7% of people in the European Union regularly exercise, while 46% of Europeans never exercise, mainly due to lack of time [39,40]. Ironically, whereas physical inactivity rises as a global public health problem, evidence on therapeutic benefits of physical activity on health continue to grow. Regular physical exercise is consensually recognized as an important preventive and/or therapeutic non-pharmacological tool against a range of metabolic, cardiovascular, musculoskeletal, pulmonary, neurological, oncological and psychiatric diseases, [36,41]. Among other mechanisms, physical exercise expresses its beneficial effects against pathophysiological conditions by targeting mitochondrial function, morphology, and bioenergetics [42], which clearly makes it a potent and interesting therapeutic stimulus against, at least, those diseases that are characterized by mitochondrial dysfunction. Our group has already reported the advantageous effects of physical exercise on mitochondriopathies related to cardiac dysfunction induced by doxorubicin (DOX) [[43], [44], [45], [46], [47]] or ischemia-reperfusion [46,47] and brain dysfunction induced by DOX [48,49], as well as those mitochondrial dysfunction associated with high-fat diet (HFD)-induced adiposopathy [50,51] and NAFLD [[52], [53], [54], [55]]. Therefore, taking into account that NAFLD is a multi-organ disease, lifestyle modifications, including physical activity, should be promoted among patients in order to impact both hepatic and extra-hepatic manifestations of this disease [10]. Furthermore, skeletal muscles act as endocrine-type organs, producing myokines during exercise, also expressing multi-organ effect targeting contractile and non-contractile tissues, including liver, adipose tissue, and brain [36], which proves the exercise as a potent protective mechanism against those multi-organ involving diseases.

## 2 Mechanisms of NAFLD development

Since 1980, when an advanced stage of fatty liver diseases, usually thought to be exclusively induced by alcohol overconsumption, was identified in people who do not consume excessive amounts of alcohol, NAFLD has been recognized as a serious metabolic disorder. At that moment, although the cause and the related mechanisms of this condition were still unclear, it was mainly identified in moderately obese patients and/or those with associated diseases such as diabetes [56].

Being considered a complex disease, NAFLD results from the interactions of many environmental and genetic factors. The PNPLA3 (patatin-like phospholipase domain-containing 3), APOB (apolipoprotein B), MBOAT7 (membrane bound O-acyltransferase domain containing 7), TM6SF2 (transmembrane 6 superfamily member 2), GCKR (glucokinase regulatory protein), and LYPLAL1 (lysophospholipase-like 1) are only some of genetic *loci* recognized as determinants in the development of NAFLD [57], while lifestyle changes, especially over-nutrition and physical inactivity, are considered main environmental culprits for NAFLD development.

Being a primary organ of glucose and lipid metabolism, the liver is particularly affected by the excessive accumulation of lipids originated from the diet, lipolysis in adipose tissue, or even *de novo* lipogenesis in liver itself, which is obviously aggravated by physical inactivity. Excessive accumulation of lipids, in at least 5% of hepatocytes, represents the first recognizable stage of NAFLD – hepatic steatosis. These accumulated lipids, in the form of micro- or macrovesicles, may cause injury to hepatocytes, leading to the advanced stage of NAFLD, known as non-alcoholic steatohepatitis (NASH), characterized also by inflammation and liver fibrosis. Patients with NASH are at higher risk to further develop cirrhosis and/or hepatocellular carcinoma (HCC) [4,58]. Despite current scientific efforts, the mechanisms of NAFLD pathogenesis remain elusive. One of the first proposed hypothesis explaining NAFLD mechanisms was the ‘two-hit hypothesis’. According to this suggestion, during the first hit, due to the imbalance in lipid metabolism and transport, the accumulation of lipids in hepatocytes occurs and leads to hepatic steatosis. Accordingly, the first hit makes the liver more susceptible to the second hit, associated with a more progressive hepatic damage. During this following stage, inflammatory cytokines are released, levels of oxidative stress increase, and a number of organelles, including mitochondria and endoplasmic reticulum, undergoes dysfunctional modifications characteristic of NASH [59]. Nevertheless, recent studies have challenged this hypothesis suggesting that multiple other “hits”, such as IR, adipokines release, nutrition (overconsumption and caloric intake), alteration in intestinal microbiota composition and activity, increased gut permeability, and (epi) genetic constraints may be involved in the pathogenesis of NAFLD and its progression from hepatic steatosis to NASH [60,61].

This ‘multiple hit hypothesis’ has also been referred to explain the development and progression of NAFLD in children and adolescents, the so-called pediatric NAFLD [62], with *in utero* environment suggested as the ‘first hit’ in the development of pediatric NAFLD [63]. The rising global prevalence of pediatric NAFLD [64,65] urges for understanding the onset mechanisms and improvement of current preventive and therapeutic approaches. Data suggest that children with family history of hepatic steatosis are at higher risk to develop this disease [66]; however unhealthy maternal lifestyles and IR during pregnancy, particularly increased in women with GDM, may also cause hepatic lipid excess in fetus. These conditions create a cytotoxic environment in the early fetal life, which “opens the door” for the development and progression of metabolic disorders [63], namely major alterations in the glucose and lipid metabolism. With advancing pregnancy, fasting glucose levels decrease while glucose production in liver increases, subsequently leading to increased fasting insulin levels and IR. Consequently, circulating glucose and free fatty acid levels increase in mothers to provide enough nutrients for fetal development [67]. However, in women who already have family history of diabetes, who are obese and/or have hyperglycemia, these metabolic alterations during pregnancy are even more exacerbated, being the levels of IR even higher and the risk to develop GDM increased. Although the impaired glucose tolerance and IR, characteristic for this type of diabetes, usually disappear after delivery, GDM carries an increased risk for the development of different metabolic disorders in both mothers and children [68,69]. In fact, as fetal metabolism cannot buffer the excess of lipids originated from maternal nutrition and transmitted through placenta [70], exposure to maternal GDM increases the susceptibility to NAFLD in children [71,72]. Moreover, despite the higher prevalence of NAFLD in men than in women in younger to middle age, a few clinical studies have shown that women with prior GDM are more likely to develop NAFLD in middle age [[73], [74], [75]].

## 3 Mitochondrial network failure in NAFLD

### 3.1 Lipid metabolism in mitochondria

Being one of the high-metabolic-rate tissues, the liver plays an important role in the metabolism of carbohydrates, proteins and lipids. However, unlike adipose tissue, the liver is not specialized in lipid storage. The pool of free fatty acids (FFAs) is maintained, on the one hand, by the balance between the lipid intake from the diet, lipolysis in adipose tissue, and *de novo* lipogenesis in the liver and, on the other hand, by the removal of liver lipids through secretion into the circulation, β-oxidation [76], or autophagy [77]. In physiological conditions, FFAs enter liver mitochondria where FFAs undergo β-oxidation or remain in the cytoplasm where they are esterified into triglycerides (TGs), which can be secreted into the circulation as very low-density lipoproteins (VLDLs). The increase of FFAs due to excess caloric intake and decreased energy expenditure associated to sedentary behavior leads to the TGs accumulation as lipid droplets in hepatocytes [25], which is characteristic for NAFLD [52,54]. Additionally, the availability of FFAs is also regulated by a selective form of autophagy, also known as “lipophagy”, which appears to have an important function in the regulation of hepatic lipid stores [77]. Excessive lipid storage, however, may inhibit autophagy, leading to a vicious cycle in which decreased autophagy increases lipid accumulation, which further impairs autophagy and promotes over-excessive lipid accumulation [78]. Indeed, decreased autophagy and associated endoplasmic reticulum stress were reported in murine genetic and dietary models of obesity [78,79], as well as in NAFLD-diagnosed patients, dietary NAFLD murine model and *in vitro* human hepatic cells [80]. The finding that autophagy is involved in breakdown of lipid droplets in liver cells suggests that autophagy impairments may have a critical role in the pathogenesis of NAFLD [78], which supports the role of physical activity as an important tool in the prevention of NAFLD through autophagy promotion [81,82].

Until recently, lipid droplets were considered simple lipid storage vesicle. However, their complex dynamic nature and functions contributed to their distinction as intracellular organelles. Lipid droplets have a neutral lipid core (TG and esters) coated by a phospholipid monolayer and specific scaffolding proteins re-localized from the endoplasmic reticulum or cytosol to their surface. The synthesis of neutral lipid begins within the endoplasmic reticulum membrane bilayer, which further deforms and lipid droplet buds forms an initial lipid droplet. These initial lipid droplets are relatively small (less than 1–100 μm) with their size and number differing among cell types [83,84]. Apart from their role in lipid storage and metabolism, lipid droplets are also involved in numerous cellular events, such as fatty acids trafficking, protein degradation, modulation of nuclear function, virus assembly and pathogen infections, and response to ER stress [83,85]. Due to these various roles of lipid droplets, aberrations in their biology, as well as, lipolysis and autophagy as main catabolic pathways of lipid droplets, are associated to many pathological conditions [86]. Moreover, by isolating lipids, lipid droplets protect cells from excessive lipid accumulation, which could eventually affect membrane integrity and lead to lipotoxicity. This way excessive fatty acids are relatively inert and harmless. However, due to imbalances in processes that maintain FFAs pool in the liver, a non-physiological accumulation of lipid results in changes not only of the number, but also the size of lipid droplets in liver cells. The hallmark of NAFLD is the formation and accumulation of small (microvesicular steatosis) or large lipid droplets (macrovesicular steatosis) in liver cells. In both cases, lipid droplets are considerably larger than those lipid droplets present in liver cells in physiological conditions [84]. The increased size of lipid droplets in pathological conditions arises from several processes: a) TG synthesis in lipid droplets, or b) fusion processes – ripening (diffusion of contents of one lipid droplet into another one) and coalescence (a true fusion of lipid droplets) [84,85]. Besides, some lipid droplets scaffolding proteins, like perilipins, might be involved in physical and metabolic interaction between lipid droplets and mitochondria, and thus in control of local fatty acid flux [87], suggesting that altered interaction between lipid droplets and mitochondria via lipid droplets scaffolding proteins might be present in pathological conditions.

### 3.2 Mitochondrial bioenergetics

Mitochondria, which occupy almost 20% of hepatocytes area, markedly contribute to the hepatocyte metabolism, especially to β-oxidation and oxidative phosphorylation [88]. Therefore, the lipid accumulation in hepatocytes is only a superficial part of NAFLD pathology. Given their key role in energy production, pH regulation, calcium (Ca^2+^) and redox homeostasis, liver mitochondria are essential sensors of cell toxicity. Mitochondria control lipid metabolism in liver; however, as in pathological conditions the liver takes over the role of lipid storage, mitochondrial dysfunction is the logical consequence. In fact, lipid accumulation in hepatocytes is known to disrupt liver mitochondrial bioenergetics, which works in a way to maintain cellular homeostasis through multiple pathways [89]. In pathological conditions, increased FFAs accumulation favors an increase of β-oxidation and subsequent generation of NADH, which serves as co-enzyme in the mitochondrial electron transport chain (ETC). However, this increased electron flux through the ETC combined with cytochrome c (Cyt c) release due, at least in part, to tumour necrosis factor-α (TNF-α) membrane permeabilization promotes an ETC impairment and an over-reduction of the respiratory chain. In these disturbed metabolic conditions, mitochondrial-related ‘electron leakage’ results in the increased ROS generation, which are able to interact with different macromolecules and affect the normal structure and function of cells and their organelles, including mitochondria themselves [1]. In addition, mitochondrial DNA is more susceptible to DNA damage by ROS than nuclear DNA as it lacks histones, and has only a limited repair capacity [90]. These enhanced oxidative stress conditions trigger the production of inflammatory cytokines, causing inflammation and fibrogenic response, and all of these, together with already extant lipid droplets in hepatocytes, lies in the heart of NASH [[91], [92], [93]].

During the initial phase of NAFLD development, liver mitochondrial bioenergetics alters and undergoes ‘remodeling’ in order to adapt to nutrients overload and protect against NAFLD progression. In this first stage, mitochondrial respiration increases, but as the disease progresses it becomes inefficient due to mitochondrial electron leakage, leading to the increased ROS production [89,94]. Among other factors [95], the failure of such an important mitochondrial adaptive mechanism is the trigger for progression from simple steatosis to NASH [89,94,96]. These alterations will depend on the progression of the disease and the ability of liver to adapt and possibly store lipids. Still, under chronic conditions, compensatory mechanisms of the liver fail to adapt, and these metabolic pathways are constrained, resulting in lipotoxicity and consequent inflammation and development of NASH [89]. Lipotoxicity-induced liver inflammation has been associated with adipose tissue remodeling and obesity-prompted NAFLD [97]. Impaired metabolic compensation in adipose tissue results in compromised energy homeostasis, and subsequently to obesity and NAFLD progression [98].

In the prolonged state of over-nutrition and excess accumulation of lipids in the liver, mitochondrial bioenergetics collapse and ATP synthesis rates significantly begin to decrease. Indeed, dysfunction of mitochondrial ETC and correspondent decrease in ATP synthesis rates are documented in NAFLD patients and animal models [76,94,99]. Ironically, the flux of TCA cycle in the liver remains elevated, probably in response to the high-energy demands in these conditions, which supplies an inefficient mitochondrial ETC with reducing equivalents and provides conditions for a vicious-cycle of increased ROS production [99].

### 3.3 Mitochondrial dynamics

Taken together, the above-referred rationale highlights that, although the mechanisms of NAFLD are still not fully clarified, it is consensually accepted that the structural and functional alterations of the mitochondrial network significantly contribute to the pathogenesis of NAFLD [100]. Being morphologically dynamic organelles, mitochondria change shape through fusion and fission-related mechanisms depending on the available energy and supply as well as the levels of stress [101]. Processes of mitochondrial fusion, fission and biogenesis are tightly controlled in response to metabolic stressors. Mitochondrial fission considers the division of one mitochondrion into two mitochondria and it is mediated by the interaction of cytoplasmic mitochondrial fission protein dynamin-like/related protein 1 (DLP1/Drp1) and other mitochondrial fission proteins, such as human fission factor-1 (Fis1), mitochondrial fission factor (MFF), and mitochondrial dynamics proteins of 49 kDa (MiD49) and 51 kDa (MiD51) [102]. Mitochondria fusion is characterized by the union of two mitochondria into one mitochondrion. Mitofusin 1 (Mfn1) and mitofusin 2 (Mfn2) mediate fusion of the outer mitochondrial membranes while optic atrophy 1 (OPA1) fuses the inner mitochondrial membranes and enable mitochondrial fusion. In normal conditions damaged and/or dysfunctional mitochondria are degraded through mitophagy in order to prevent a release of “toxic” signals from mitochondria into the cytosol. Partly damaged mitochondrion undergoes mitochondrial fission where it is divided into two smaller mitochondria – a healthy one and a damaged one. The damaged mitochondria are further degraded by mitophagy, while healthy mitochondria may eventually fuse contributing to a healthy mitochondrial pool [103].

Mitochondrial dynamics plays an important role in the bioenergetics adaptation to cellular metabolic demands. With alterations in nutrient availability, mitochondria will undergo fusion or fission in order to maintain mitochondrial energy production and bioenergetics [104]. In a rich-nutrient environment, mitochondria fragment to avoid energy waste, by decreasing bioenergetic efficiency and increasing mitochondrial uncoupling, which will eventually lead to the increased nutrient storage [104,105]. During high levels of cellular stress or when damaged, mitochondrial network undergo fission to separate damaged mitochondria from a healthy cellular content and maintain cellular and mitochondrial bioenergetic homeostasis [101]. DLP1/Drp1 mediates mitochondrial fission and some studies show increase in protein expression in animals models of NAFLD [[106], [107], [108]]. In starvation, mitochondria tend to fuse in order to maintain ATP production and, thus, increase bioenergetics efficiency. Mitochondrial elongation during starvation protects cells from autophagy and cell death [104,109]. In the presence of functionally damaged mitochondria, mitochondrial quality control mechanisms are activated and apart from potentially mitophagy-related outcomes, mitochondria may fuse to share RNAs or proteins and compensate for the functional defects. The reduced signaling for mitochondrial fusion detected in NAFLD animal models underlines its potential role in the pathology of NAFLD [106,107]. When mitophagy pathways in hepatocytes are altered, damaged mitochondria accumulate, leading eventually to the release of mitochondrial damage signals into the cytosol resulting in inflammatory cytokines expression and inflammasomes activation, and eventually development of steatohepatitis [103]. Additionally, mitochondrial dynamics is also associated with excessive ROS production and enhanced oxidative damage, therefore prompting mitochondria as a target of oxidative stress levels in metabolic diseases [108,110].

### 3.4 Mitochondrial permeability transition pore

Once mitochondrial functioning starts failing, their role in Ca^2+^ homeostasis also becomes endangered. Ca^2+^ is recognized as an important factor in the regulation of mitochondrial function. Adequate levels of cytosolic Ca^2+^ are referred as important mediators in the activation of some of the enzymes involved in oxidative phosphorylation. Furthermore, data suggest that extramitochondrial Ca^2+^ is also involved in supplying oxidative phosphorylation with substrates on demand [111,112]. However, in pathological conditions, disrupted Ca^2+^ homeostasis activates mitochondrial buffering ability to uptake it and leads to the overaccumulation of Ca^2+^ in mitochondria, which further affects mitochondrial redox homeostasis and ATP production. Moreover, Ca^2+^-related involvement in the activation of complex-like pores - mitochondrial permeability transition pores (mPTP), induces the loss of inner mitochondrial membrane permeability. As a consequence, the induction of mPTP stimulates both mitochondrial dysfunction and the release of proapoptotic proteins from mitochondria to the cytosol resulting in the activation of apoptotic-related signaling [113], and eventually of cellular quality control mechanisms, such as autophagy [114,115]. Accordingly, the activation of the mPTP is considered an important factor in the pathogenesis of fatty liver diseases, with susceptibility to mPTP opening significantly increased in animal models of NAFLD [53,116,117]. Together with other typical metabolic disturbances observed in NAFLD conditions, namely the Ca^2+^ overload and enhanced oxidative stress [116], it seems that high fat and glucose fluctuation may also trigger mPTP, leading to the unregulated flux of molecules into the mitochondrial matrix and, eventually, to mitochondrial swelling, which jeopardizes transmembrane potential and induces the collapse of mitochondrial bioenergetics [118].

### 3.5 Estrogen signaling and mitochondrial function

Another interesting issue associating NAFLD to impaired mitochondrial function are low levels of estrogen [119]. In fact, estrogen signaling, mediated by estrogen receptor α (ERα), seems to affect mitochondrial function by modulating ATP production, mitochondrial membrane potential, mitochondrial biogenesis, and Ca^2+^ signaling. ERs appear to translocate and to be transitionally present in mitochondria, where they can bind to an estrogen response elements (ERE)-like element of the mitochondrial genome and induce the transcription of mitochondrial DNA genes [120]. The protective effect of estrogen against the risk of developing NAFLD in women has already been proposed [15], while the significance of ERα in insulin signaling has been noted. Data suggest that ERα knockout mice express impaired glucose tolerance and IR, downregulation of peroxisome proliferators-activated receptors (PPAR), PPARα and PPARΔ, and uncoupling protein-2 (UCP2) expression in skeletal muscle as well as elevated inflammatory signaling in liver [121]. Considering that ERα can be co-activated by peroxisome proliferators-activated receptor-gamma coactivator 1-α (PGC1α), the loss of estrogen signaling, present in postmenopausal women, is possibly linked to reduced PGC1α and associated with liver fat and NAFLD progression, which may explain, at least in part, gender-specific difference in NAFLD development and the increased risk for NAFLD in women after menopause. Furthermore, the loss of estrogen signaling can also exacerbate diet-induced NASH in a rodent model, which highlights the impact of this hormonal-mediated effect in the progression of this pathological condition [122]. Ultimately, an epigenetics-mediated regulation may also explain some of the possible mechanisms that associate estrogen with protective effects against NAFLD risk in women. In a study performed by Zhang at al. [123] and devoted to better understand the role of estrogen signaling in liver of male and female mice diagnosed with NAFLD, *in vivo* and *in vitro* experiments showed that the upregulation of miR-125b might explain, at least in part, the protective mechanism associated to estrogen. The miR-125b, activated by estrogen through ERα, targets fatty acid synthase and possibly alter lipid metabolism in a way that protects against NAFLD [123].

## 4 Physical (in) activity-related NAFLD

Being NAFLD one of the most prevalent liver diseases in western countries, the questions of developing effective preventive and/or therapeutic countermeasures against the disease emerge. A design of effective pharmacological treatments was the first starting point, usually including drugs targeting associated metabolic disorders, such as diabetes and IR or pharmacological strategies used in the prevention of mitochondrial degeneration, which is also present in NAFLD. Some of these strategies also included mitochondrial-targeted molecules, antioxidants administration, and anti-obesity drugs; however, all of them showed low levels of effective therapeutic efficiency. Therefore, the US Food and Drug Administration (FDA) has not approved any of them in the treatment for this disease. So far, weight reduction and lifestyle modifications (dietary changes and physical activity) are still the only ones proposed as effective interventions for the treatment of NAFLD [124,125].

### 4.1 Physical exercise to prevent NAFLD

Despite the fact that the knowledge regarding the effects of physical exercise on non-contractile tissues is emerging, a research in the area of NAFLD treatment with exercise is still scarce. However, a number of studies with NAFLD patients evidence the importance of lifestyle changes in dealing against this disease [[126], [127], [128]]. Several clinical studies suggest a positive association between sedentary time and the prevalence of NAFLD. Using subjective (questionnaires) or objective (motion sensors) methods to assess physical activity, several studies report that patients with NAFLD spend more of their daily time in sedentary activities than healthy people. In these NAFLD patients, body mass index and weight are positively associated with sedentary lifestyle. Thus, it is not a surprise that the number of steps per day, total daily energy expenditure, and metabolic equivalences (METs) are lower in NAFLD patients than in healthy subjects. Furthermore, these studies showed that higher-intensity exercise is associated with lower prevalence of NAFLD [[129], [130], [131], [132]]. Therefore, increased physical activity seems to be able to prevent the development and progression of NAFLD through different pathways, including the increase of energy expenditure by muscle contractions and through the stimulation of glucose uptake from the circulation into the muscles [131]. Skeletal muscle is the primary organ of disposal of glucose, as the response to insulin. Therefore, the loss of muscle mass, might be involved in the development of IR and IR-related metabolic disorders, including NAFLD. On the other hand, sarcopenia, an aging-related loss of muscle mass, can also occur earlier in life due to lifestyle changes, particularly unhealthy diet and physical inactivity, which are also in the background of NAFLD development [133]. Thus it seems that sarcopenia and NAFLD share similar risk factors and underlying mechanisms. By improving energy balance and insulin sensitivity, physical activity can contribute to the reduction of hepatic lipid accumulation. Better understanding of liver–muscle interaction could improve the management of NAFLD [134].

Taken together, data suggest that increased physical activity is an effective protective non-pharmacological strategy against several metabolic disorders and a therapeutic approach for patients diagnosed with NAFLD. Indeed, regular exercise has been shown to improve liver histological and metabolic features in NAFLD patients. Studies in both NAFLD patients [[126], [127], [128]] and animal models of NAFLD [52,54,55,135] indicate that physical activity is effective in reducing lipid content in hepatocytes and enhancing insulin sensitivity. Although the underlying mechanisms of the protective effects perpetrated by physical activity still remain elusive, some of them may rely on the inter-organ pathways established between liver, skeletal muscles, and adipose tissue. At a glance, skeletal muscles are the main disposal sites for the glucose in post-prandial state. Therefore, by increasing the level of glucose uptake by the muscles and the activity of glycogen synthase in myocytes, the peripheral insulin sensitivity is improved [136]. Also, regular physical activity reduces visceral adiposity in NAFLD patients [126,137], which contributes to lower the influx of fatty acids from visceral adipose tissue to the liver and prevent possible liver lipid accumulation [126].

### 4.2 Physical exercise during pregnancy

As previously referred, GDM enhances the risk of metabolic impairments in the offspring, but also, an increased likelihood for the development of NAFLD in mothers with GDM sooner than it is expected in non-pregnant women. Considering this transgenerational burden, strategies that mitigate the development of GDM need to be seriously implemented in order to protect both mother and fetus from this deleterious condition, being physical exercise a non-pharmacological approach that is becoming more and more exciting. Taking into consideration that in uncomplicated pregnancy, there are no impediments to engage in exercise programs and it is considered safe and beneficial during pregnancy, the American College of Obstetricians and Gynecologists (ACOG) recommends that healthy women or even athletes, after the consultation and clinical evaluation by obstetricians/gynecologists, continue to regularly exercise, although some adjustments should be made on the exercise programs and training routines due to the anatomical and physiological changes associated to pregnancy. In fact, non-athlete pregnant women are encouraged to perform at least 30 min of moderate intensity physical exercise every day or, at least, most days of the week [138]. In women diagnosed with GDM, moderate- or high-intensity aerobic or resistance exercise, at least three times per week, lowers fasting blood glucose levels and glycated hemoglobin, and also improves glycemic control [139]. Moreover, it is known that physical exercise in pregnancy can improve physical fitness, cardiovascular function, muscle mass, weight management, sleep quality, and psychologic well-being, as well as, reduce the risk of developing GDM [140]. Therefore, it is expected that exercise may reduce the risk of maternal, fetal, and neonatal complications in GDM that could aggravate hepatic glucose and lipid metabolism and prompt development of NAFLD in both generations.

A deeper look into cellular mechanisms sheds a light on possible effects of physical activity in the prevention and treatment of NAFLD through modulation of function and structure of particular organelles, namely mitochondria.

## 5 Physical exercise and liver mitochondrial adaptations

Depending on the exercise type, intensity and duration, the predominance of substrates utilization may vary during exercise. Generically, the main substrates during low-to moderate-intensity exercise are FFAs and glucose, while with the increase of intensity, glucose becomes a more prominent fuel source, which challenges the energy homeostasis of muscles in several circumstances [42]. Considering the importance of mitochondria in cell metabolism, the effects of exercise in mitochondria are intensively studied. Although it is expected that exercise-related adaptations mainly affect muscle mitochondria, liver mitochondria are also a target during exercise. Given the role of liver in maintaining glucose and lipid metabolism, liver mitochondria are essential for producing energy for these energy-demanding processes. Therefore, as a stimulus for liver mitochondria to produce ATP to maintain homeostasis of those metabolic processes, exercise *per se* also activates systemic pathways that indirectly modulate liver mitochondria bioenergetics, function, and structure [42,141]. In fact, during exercise-related contractions, skeletal-muscle fibers can produce a number of muscle-derived molecules, the so-called myokines that are secreted into the bloodstream and target different non-contractile tissues, such as liver, brain, and adipose tissue. Apart from many other potential pathways, the myokine interaction with tissue-specific receptors on those tissues, modulates molecules that ultimately may activate PGC-1α, resulting in an improved regulation of mitochondrial biogenesis [[126], [127], [128]]. Still, the mechanisms are rather obscure. Given the known deleterious consequences of distinct pathological conditions on mitochondrial bioenergetics and dynamics, including in the liver, and the health-related relevance of maintaining an adequate mitochondrial network function and structure for cell functioning, the question whether and how exercise can positively modulate liver mitochondria, and thereby counteract adverse impact of malnutrition and sedentary behavior in the development and progress of NAFLD arises.

So far, there are no consensual suggestions regarding how physical exercise can impact liver mitochondrial signaling pathways in the animal models. Data from several research groups confirm the positive relevance of active lifestyles in the adaptation of liver mitochondria against the harmful consequences of NAFLD [[52], [53], [54], [55],^82^,^135^,^142^,^143^]. In fact, two models of exercise (voluntary physical activity (VPA) performed in free-running wheels and endurance training (ET) in the treadmill) in animal models were able to mitigate impairments in mitochondrial bioenergetics and dynamics present in a NASH model induced by a HFD. Both exercise modalities were able to positively modulate liver mitochondria bioenergetics function, reflected by the beneficial changes in the respiratory control ratio [54]. The preservation of liver mitochondrial function can, at least in part, be explained by improvements of mitochondrial membrane structured in the exercise groups. The ratio between phosphatidylcholine and phosphatidylethanolamine, as a marker of membrane integrity, was reduced in sedentary HFD group, suggesting an impaired membrane integrity that was reverted by physical exercise. Nevertheless, cardiolipin, which plays a main role in mitochondrial bioenergetics, decreased in both sedentary and exercise HFD groups [52]. Alterations in the mitochondrial membrane permeability and the induction of mPTP lead to the consequent release of proapoptic molecules and, ultimately, the progression of both the apoptotic and autophagic signaling [114,115]. These results highlight the relevance of active lifestyle strategies to reduce the susceptibility of liver mitochondria to permeability transition pore opening and to positively modulate apoptotic and autophagic signaling in animal models of NAFLD [53]. Even tough, it is important to note that, in some circumstances related with the severity of the deleterious consequences, physical exercise may also stimulates autophagy in various tissues, including liver [82,144], suggesting that one of the mechanisms through which exercise could induce its beneficial effects is the activation of the autophagic signalling [81].

Regarding the positive modulation of physical exercise on liver mitochondrial network remodeling, a study designed by Santos-Alves et al. [145] comparing two chronic exercise modalities (VPA and ET) during 12 weeks in rat model, showed that both models of exercise were able to positively alter the expression of proteins associated with mitochondrial biogenesis and dynamics and increase liver autophagy signaling, thus inducing adaptive remodeling of liver mitochondrial network. On the other hand, another study showed that 5 weeks of endurance treadmill training *per se* was not a sufficiently strong physiological stimulus to significantly alter liver mitochondrial function or protein expression of OXPHOS subunits. Still, endurance training was able to counteract the deleterious impact induced by salicylate and benefit liver mitochondrial function in the context of toxicity [146]. In contrast, an elegant study designed by Fletcher et al. [142] suggests that 4 weeks of endurance training increases OXPHOS activity mediated by complex I-related substrates although without significantly modulating liver mitochondrial biogenesis markers. In accordance, other studies suggest changes in the activity of liver mitochondrial ETC complexes in response to exercise, particularly of complexes I, IV, and V, followed by increase in mitochondrial glutathione content, and without increased ROS production [147]. Likewise, 6 weeks of swimming exercise positively modulated oxidative stress in rats’ liver mitochondria by increasing the activity of the antioxidant system and concomitantly decreasing the levels of oxidative stress [148]. Taking together, the increased antioxidant defense and decreased oxidative stress observed in the referred studies suggest that physical exercise is effective in managing ROS production during exercise and in preserving liver mitochondria redox homeostasis [147,148]. In Otsuka Long-Evans Tokushima Fatty (OLETF) rats, it was shown that, even though both aerobic exercise and treatment with metformin, usually used for the treatment of type 2 diabetes, were effective in modulating NAFLD phenotype regarding fatty acid metabolism and liver mitochondrial function, exercise training *per se* was even more effective, while the combination of drug treatment and a lifestyle change had only a slight synergistic effect [149]. Even though the general perspective highlights a positive impact of physical exercise in liver mitochondrial function and dynamics, some of the discrepancies among these studies may be related to the differences in animal species and strains used and in the features of the distinct exercise models, namely the distinct type, duration, and intensities of exercise programs [24].

## 6 Epigenetic perspective of NAFLD and lifestyle modifications

Although the precise underlying mechanisms linking lifestyle changes and development of NAFLD are not completely understood, epigenetic regulation of genes associated with NAFLD development and progression recently came to the fore [150]. Expression of microRNAs (miRNAs) as well as DNA methylation and histone modification by altering DNA accessibility, regulate the activity of genes involved in, among others, lipid metabolism, mitochondrial dysfunction, oxidative stress, and inflammation. In fact, studies on liver biopsy samples from patients diagnosed with NAFLD confirmed the alterations in methylation of genes related to lipid metabolism and mitochondrial biogenesis. Furthermore, the observed differences in DNA methylation status may help to distinguish patients with mild and advanced stages of NAFLD and can be useful to predict progression of NAFLD [151,152].

Despite of increasing evidence regarding the contribution of epigenetic mechanisms in the pathogenesis of different diseases, the role of miRNAs, DNA methylation, and histone modification in NAFLD pathogenesis is still not clear. Global screening of circulating miRNA expressed in liver under NAFLD conditions showed that the up-regulation of miR-122, mir-34a, miR-15b, and miR-16 in circulation were positively associated with NAFLD-related evidence. Increased circulatory levels of miR-122, miR-192, and miR-375 could be proposed as clinical markers of NAFLD progression from hepatic steatosis to NASH. Among these miRNAs, it seems that increased circulatory miR-122, the most abundant miRNA in the liver with an important role in lipid metabolism, has a high impact in NAFLD pathogenesis [153]. Furthermore, NASH was associated with decreased liver expression of miR-122 in NAFLD patients [154]. Similarly, the liver expression of miR-122 was downregulated, together with miR-27 and miR-451, while miR-200a, miR-200b, and mir-429, were upregulated in the liver of a diet-induced rat model of NAFLD [155]. This inverse correlation between circulatory and liver levels of miRNAs may explain their influence in the pathophysiology of NAFLD. Liver lipid-responsive miRNAs miR-34a, which targets one of the members of sirtuin family of NAD-dependent histone deacetylases, is also shown to be upregulated in NASH [154] apparently affecting histone modifications in liver. In addition, histone modifications and DNA methylation regulate gene activity and transcription. Among several transcription factors involved in *de novo* lipogenesis in liver and contributing to liver steatosis, PPAR-γ [156] and its coactivator PGC-1α drew attention in epigenetics research associated with NAFLD and its progression [[156], [157], [158], [159]]. The involvement of PGC-1α relies in mitochondrial biogenesis, β-oxidation, and lipid transport [158,160] and it's reduction in liver is associated with NAFLD progression in murine model of NAFLD [161]. It is also an important factor for expression of mitochondrial transcription factor A (TFAM) [160], which plays an important role in replication and gene expression of mitochondrial DNA [143]. Hypermethylation of *PPARGC1A*, but not of *Tfam* promoter, was detected in liver biopsy specimens from NAFLD patients [157].

Given a potency of epigenetics in diseases’ pathophysiology, new emphasis is being placed on the influence of epigenetic modifications at the mitochondrial level. miRNAs detected in liver mitochondria may act as sensors of mitochondrial environment leading to the modulation of mitochondrial function and cell signaling [162,163]. Even though, miRNAs regulation is usually related to the cytosol, it seems that mitochondria have a unique pool of miRNAs, independent of total miRNA pool in the cell. The expression of some of these mitochondria-specific miRNAs was increased in liver mitochondria of diabetic mice, such as miR-202–5p, miR-134, miR-155, as well as, miR-494, which affects *Tfam* expression and thus has a role in regulation of mitochondrial DNA [163]. Mitochondrial miRNAs are involved in regulation of transcription, gene expression, gene silencing, cell cycle and cell division, chromatin modification, and *in utero* embryonic development [164]. Additionally, methylation of mitochondrial DNA has been proposed as a biomarker in disease diagnosis, considering that this epigenetic mechanism may be involved in the pathophysiology of some diseases. However, due to the specificity and structural organization of mitochondrial DNA, which lacks in histones, the methylation of mitochondrial DNA may also be a very specific mechanism and further investigation is necessary. So far, some studies have been conducted in areas of drug treatment, oxidative stress, aging, and neurodegenerative diseases [165]. Still, in liver mitochondria of NAFLD patients, methylation of mitochondria encoded NADH dehydrogenase 6 was higher in NASH patients when compared to hepatic steatosis patients [166], suggesting a role of epigenetic alterations not only on nuclear DNA, but also on mitochondrial DNA in the development and progression of NAFLD.

The emerging epigenetic research seems to explain possible underlying mechanisms in NAFLD pathogenesis, but also provides new surprising perspectives on how epigenetic mechanisms play a role in offspring programming. In fact, maternal nutrition has been associated with epigenetic alterations in key genes involved in lipid metabolism and accumulation, such as *Ppara*, *Ppargc*, and *Fgf21*, suggesting that altered DNA methylation patterns in fetal development due to maternal lifestyles may influence offspring health later in life [22,23]. In rodent models, offspring of mothers fed with HFD had altered gene expression, inhibition of hepatic cell cycle and associated DNA methylation [167,168]. *In utero* exposure to maternal HFD affected the genes related to liver dysfunction, immune response, and inflammation, and led to DNA hypermethylation of the genes. Furthermore, these epigenetic alterations, some of them persistent through the life, were associated with prolonged gene expression alterations in the offspring liver, possibly enabling the development of metabolic dysfunctions later in life [169]. Moreover, maternal GDM impacts the epigenome of their offspring, mainly through metabolic pathways affecting fetal growth and development, suggesting that DNA methylation is one of the mechanisms mediating fetal programming. The exposure to GDM in early fetal life is associated with DNA methylation alterations in the genes involved in metabolism, endocrine function, and insulin signaling [170,171].

Ultimately, considering the above-mentioned relevance of epigenetic regulation in the development of NAFLD, epigenetic-related analysis regarding the impact of physical exercise in liver mitochondrial function and dynamics will be, for sure, a future emerging and growing field of study. Some studies propose DNA methylation status as a marker of progression of NAFLD [151,152], however, this field remains quite open for research regarding NAFLD treatment through changes in lifestyle. Presently, the modulation of the epigenome by changes in lifestyle seems to be able to revert the adverse effect of malnutrition and sedentary lifestyle. Zhou et al. [172] showed that fast food diet increased liver epigenome susceptibility to develop metabolic diseases. On the other hand, physical exercise was able to protect against this fast food diet-induced DNA hypermethylation [172]. Still, most of the studies address the modulating effect of exercise on skeletal muscles epigenome rather than in the liver. For instance, acute exercise was able to decrease promotors methylation of genes involved in mitochondrial biogenesis, PGC-1α, PPAR-γ, and TFAM in soleus muscles, which suggest that DNA hypomethylation is an early event in contraction-induced gene activation for structural and metabolic adaptations in exercised skeletal muscles [173]. Furthermore, circulating miRNAs can be altered as a physiological response to physical exercise. For instance, in muscle and heart, mir-486 may affect glucose metabolism, while mir-499 is involved in mitochondrial metabolism and apoptosis through Drp1-related signaling. The better understanding of the effect of physical activity on specific circulating miRNAs could justify the use of exercise as a therapeutic strategy [174]. In a HFD-induced animal model of NAFLD, exercise was able to reduce hepatic overexpression of mir-212, which appeared to be involved in lipogenesis and development of NAFLD by targeting FGF-21 [175]. In the study involving HFD-induced obesity in rats, hypoxic training reduced hepatic levels of mir-378, involved in the regulation of lipid metabolism and production of TGs, and, subsequently, increased energy expenditure, β-oxidation in rat liver, and obesity-related morphological and biochemical features. The study also suggests that the probable mechanism is through muscle utilization of fatty acids produced in the liver [176].

In this regard, it is important to note that not only nuclear DNA, but also mitochondrial DNA may undergo epigenetic modifications that are associated with the development of several diseases. In fact, mitochondrial DNA can also be methylated, which attracts attention in the field of disease treatment and prevention. In NASH patients, the methylation level of NADH dehydrogenase 6 encoded by mitochondrial genome was inversely correlated to the level of physical activity [166]. Still, data are scarce and mitochondrial epigenetics and its association to disease risks are still to be elucidated. Therefore, the clarification of the impact of exercise-related epigenetic mechanisms on cellular processes may contribute to this emerging field and promote active lifestyles.

## 7 Concluding remarks

The prevalence of NAFLD and related metabolic disorders (metabolic syndrome, insulin resistance, obesity) is increasing worldwide. Despite numerous pharmacological therapeutic approaches used to counteract multi-organ manifestation of NAFLD, which appear as highly ineffective, physical exercise emerges as a beneficial non-pharmacological preventive and/or therapeutic strategy against this disease. Physical exercise has already been shown as beneficial against some pathological features characteristic for metabolic liver dysfunction, and especially mitochondrial dysfunction. Additionally, considering that the lifestyle changes of parental generation may influence metabolic status of their offspring, widely known benefits of physical exercise, particularly during pregnancy period, should be taken into account. The use of physical activity during pregnancy as a preventive tool against developing NAFLD in mothers and even against transmitting their impaired metabolic burden to offspring is challenging, being that the mechanisms behind the supposed resistant phenotype are so far not studied. Bearing in mind the potency of epigenetic mechanisms in the development of NAFLD and the transmission of those metabolic impairments to future generation, better understanding of epigenetic-related impact of physical exercise on liver mitochondrial function will be a determining factor in the design of beneficial exercise programs in the future. The further mechanistic look of how physical activity interacts with hepatic mitochondrial “fitness”, particularly via epigenetic mechanisms, may provide necessary clues for proposing active lifestyles during pregnancy as the important preventive strategy to reduce the risk of liver injury through generations. Additionally, a better understanding of muscle–liver axis is expected to delineate mechanisms by which exercise offers benefits in the prevention and treatment of liver diseases.
